# Interpersonal synchrony: Interaction variables and gender differences in preschoolers with ASD

**DOI:** 10.3389/fpsyt.2022.1009935

**Published:** 2022-09-14

**Authors:** Eleonora Paolizzi, Giulio Bertamini, Arianna Bentenuto, Paola Venuti

**Affiliations:** ^1^Laboratory of Observation, Diagnosis and Education (ODFLab), Department of Psychology and Cognitive Science, University of Trento, Trento, Italy; ^2^Data Science for Health (DSH), Bruno Kessler Foundation (FBK), Povo, Italy

**Keywords:** autism spectrum disorder, females, interpersonal synchrony, gender differences, interaction variables

## Abstract

**Background:**

Females with ASD tend to be under-recognized as they might present a different symptom manifestation, better social abilities, and masking behaviors. Since the main limitation of current literature on gender differences is represented by focusing on broad constructs, research needs to prioritize narrower constructs related to the subdomains of social abilities. Hence, the aim of this work was to explore gender differences in Interpersonal Synchrony of children with ASD.

**Method:**

*N* = 51 psychologist-child dyads, 25 females and 26 males participated in the study. An Observational Coding Scheme to study interaction features was applied to video-recorded sessions of the ADOS-2 administration.

**Results:**

Females presented more synchronous behaviors with shorter latencies. Their interplays were longer, more complex, more engaging and most frequently adequately concluded with respect to males. The complexity of interchanges, their total duration and the proportion of exchanges adequately terminated correlated with the Social Affect score, Personal-Social, and Language Quotients in females, but not in males. The success rate of psychologist proposals correlated with Language Quotient in both males and females. The number of exchanges positively correlated with the Performance Quotient in males. Despite females being significantly older than males, age-related differences did not emerge.

**Conclusions:**

Our findings suggest the importance of studying gender differences with respect to interaction variables. Females may present better IS abilities which, in turn, may promote social and language development. Further, our results suggested that successful interactions seem to rely more on social abilities in females, while males appeared to rely more on performance skills.

## Introduction

Autism spectrum disorder (ASD) is a neurodevelopmental disorder characterized by core symptoms in social communication and interaction, together with patterns of repetitive, restricted behaviors, interests and activities (1). Although previous research consistently showed a male predominance, with ASD being four times more frequent in boys, recent estimates highlighted lower male-female ratios of 2:1 or 3:1 (2–4). However, girls still tend to be diagnosed later in life (4). Several reasons have been suggested for the under-recognition of females, among them: different symptoms manifestation (5), compensatory and masking behaviors, also referred to as “camouflage” (6, 7), and differences in social and communication skills. In particular, females may present better eye contact, facial expressions (8), better imitative abilities (3, 9). Additionally, they might appear more reciprocal (10) and show relatively preserved pretend play skills (10–12). Further, males with ASD spend less time jointly engaged during interplays and more time in solitary play, when compared to females (13). Researchers also reported better communicative skills in girls with ASD compared to boys (14), with lower difficulties in reestablishing a conversation, a more flexible use of language (15), and better eye contact (16). Researchers should consider better investigating the relation between clinical measures and the relative abilities, employing observational tools to better understand females under-recognition. This is particularly relevant considering also the influence of gender norms, since more passive behaviors are generally more accepted when displayed by females (3, 17). Unsurprisingly, teachers tend to report less concern related to the social behaviors of girls with ASD (10).

Research on gender differences is still at an initial phase and studies are mainly conducted investigating broad constructs. This raises the need of narrowing the focus down, particularly to the subdomains of social abilities (18). For this, Interpersonal Synchrony (IS) could be considered to deepen the knowledge of gender specific interaction profiles, and ultimately to understand why females with ASD tend to be diagnosed later than males. IS is a measurable construct related to intersubjectivity and it can be studied from birth (19). It refers to a dynamic process (20) characterized by the multimodal and temporal coordination of verbal and non-verbal, communicative and emotional signals of two communicative partners during a social interaction (19–21). IS plays a critical role in the social, emotional and self-regulation development (19–23). Social, and cognitive aspects, specifically memory (24, 25) and language development (20, 26), appear to be influenced and scaffolded by means of IS. Given the importance of synchrony in human development, as shown by studies on the neurotypical (20, 23, 27, 28) and ASD samples (19, 21, 29), the paucity of literature on gender differences concerning this construct might hinder researchers' ability to understand gender differences more broadly.

To the best of our knowledge, researchers showed that children with ASD present impairments in IS when compared to non-autistic ones (21, 29), but gender differences have never been explored before.

The aim of this work was to explore gender differences in IS of preschoolers with ASD while interacting with a psychologist in a semi-structured environment, using a quantitative observational measure. In fact, a limitation of previous studies is represented by the fact that they were mainly conducted using instruments not standardized on female samples (3). Thus, we employed a more objective measure to disclose interactional gender differences.

Considering that recent research suggested that females with ASD might present better social skills and that their development is strictly connected to IS, IS may be a relevant construct when studying gender differences. Based on current literature on IS and about gender-based differences in ASD, our exploratory hypotheses were the following.

We expected that females might present (a) more synchronous behaviors with (b) longer, and (c) more engaging interplays. These hypotheses were based on current literature showing that females are perceived as more reciprocal by adults (10) and spend more time engaged when playing with peers (13).

## Materials and methods

### Participants

*N* = 51 psychologist-child dyads, 25 preschool females (mean age = 48.560, sd = 13.863) and 26 preschool males (mean age = 38.962, sd = 10.375) participated in this study.

The sample was selected based on the following inclusion criteria from a dataset of clinical data at the Laboratory of Observation Diagnosis and Education (ODFLab) of the Department of Psychology and Cognitive Science of the University of Trento:

a) A diagnosis of ASD defined following the Diagnostic and Statistical Manual of Mental Disorders—5 version criteria (1). It was carried out by licensed psychologists of the Laboratory of Observation Diagnosis and Education (ODFLab), a clinical research center of the University of Trento (Italy), specialized in functional diagnosis of neurodevelopmental disorders.
b) No comorbidities with other psychiatric conditions.
c) Subjects with an age between 18 and 84 months.
d) The availability of the first ADOS-2 administration video-recording.

### Measures

#### Observational coding scheme

We employed an observational coding system to quantitatively study the bidirectional interaction patterns displayed by the child and the clinician during the exchange (30). The coding schema consists of 15 codes that individuate both adult and child behaviors.

Interactions can be defined based on two scenarios: Interaction Units and Shared Actions. The Interaction Units refer to the pairs of consequent child and therapist behaviors', defined by point events. The annotated child behaviors are those characterized by intentionality and social motivation, while the ones related to therapist's behaviors aim to measure his responsiveness and sensitivity. Specific Interaction Units can lead to a sharing: the therapist proposal (TP), possibly accepted by the child (CA), the child proposal (CP) accepted by therapist (TA), and the child intentionality signal (CI), possibly caught by therapist (TI). Other behaviors' sequences can be coded when the child shows signs of dysregulation (CD), recognized by the therapist which adapts his behavior to the child's state (TR). Further, therapist proposals (TP) can also be actively refused by the child (CR), or by child indifference. In this latter case nothing is coded.

The coding system also measures the mutual engagement of the dyad. After a successful unit of interaction that initiates a social routine, the code Shared Activity (SA) describes a state event that measures interactions' duration. Further, each interplay is characterized by the mean level of engagement displayed by the child (ENG), which is evaluated on a three-point scale from lowest (1) to highest (3) considering the level of participation and shared pleasure shown by the child. Exchanges are also characterized by complexity, defined by the number of sequences Therapist Widens (TW) followed by child response. TW can be found when the psychologist tries to raise the level adding new requests, introduces variations to prevent the establishment of a rigid behavioral pattern or reduces the level of stimulation to keep the child engaged. Finally, a Shared Activity is also defined by how it ends, as well as by who: it can be terminated by the therapist, independently from the child's agreement (TE). Further, it can be adequately concluded by the child, considering its developmental level and communicative abilities (CE), or by child withdrawal from the interaction, by “getting stuck” into repetitive behaviors with no social meaning, or suddenly moving away from the therapist (CX) (see Table 1).

**Table 1:** Coding schema of the observational code and interclass correlation coefficient for each code (30).

**Code**	**Description**	**ICC (alternative hypothesis r0 > 0.8)**
TP*	Therapist proposes	0.958; *F*_(9, 5.100)_ = 5.400; *p* = 0.038 [0.764–0.990]
TW*	Therapist widens	0.953; *F*_(9, 9.090)_ = 4.620; *p* = 0.016 [0.824–0.988]
CA*	Child accepts	0.952; *F*_(9, 5.580)_ = 4.600; *p* = 0.039 [0.765–0.989]
*CR*	Child refuses	0.627; *F*_(9, 9.49)_ = 0.477; *p* = 0.858 [0.096–0.889]
CI*	Child's intentionality	0.940; *F*_(9, 7.990)_ = 3.640; *p* = 0.042 [0.766–0.985]
TI*	Therapist recognizes intentionality	0.957; *F*_(9, 5.820)_ = 5.250; *p* = 0.030 [0.786–0.990]
*CP*	Child proposes	1
*TA*	Therapist accepts	1
*SA*	Shared activity	1
CX***	Child inadequately ends the sharing	0.992; *F*_(9, 10)_ = 28.800; *p* <0.001 [0.971–0.998]
*TE*	Therapist ends activity	1
CE**	Child adequately ends the activity	0.975; *F*_(9, 10)_ = 8.700; *p* = 0.001 [0.908–0.994]
*CD*	Child's signals of dysregulation/acrtivation state	0.571; *F*_(9, 9.870)_ = 0.401; *p* = 0.907 [0.014–0.869]
*TR*	Therapist recognizes child's dysregulation signals	0.667; *F*_(9, 9.960)_ = 0.556; *p* = 0.805 [0.138–0.904]
ENG 1 (low engagement)	Child engagement displayed during the interplay	0.943; *F*_(9, 5.91)_ = 3.9; *p* = 0.057
ENG 2 (medium engagement)		0.741; *F*_(9, 6.56)_ = 0.738; *p* = 0.672
ENG 3 (high engagement)		1

*p <0.05; **p <0.01, ***p <0.001.

The Observational coding schema has previously shown a satisfactory construct validity. Further, two coders, experts in the field of autism spectrum disorder, were trained by a researcher to a reliability criterion of α > 0.80 and have previously shown satisfactory inter-rater reliability, measured with the Intraclass Correlation Coefficient (ICC) on the 20% of the videos independently coded (*n* = 10) (30) (For further information, see Table 1).

#### Autism diagnostic observation schedule—Second edition

The Autism Diagnostic Observation Schedule-2 (ADOS-2) (31) is a semi-structured observational instrument considered as a golden standard for the diagnosis of ASD and the administration is carried out by trained psychologists who participated to an official ADOS-2 course that allows the reliable use of the tool in clinical and research practice. It evaluates the two main areas of impairments defined in the DSM-5 criteria: Social Affect and Restricted, Repetitive Behaviors areas. It consists of five modules, based on the person's chronological age and level of expressive language. In this study, modules Toddler, 1, 2, and 3 have been used. For each module, the Social Affect (SA) and the Restricted Repetitive Behaviors (RRB) scores can be calculated by means of an algorithm that considers specific sets of observational items. Higher scores in the two domains indicate a greater presence of ASD symptoms. Further, a Total Score determines whether the behavioral manifestation examined falls in the autism-autism spectrum–non spectrum classification. Finally, it can be converted to a Comparison Score to determine the severity of the symptoms.

#### Griffiths mental development scales—Edition revised

The GMDS-ER (32) is a semi-structured instrument to assess child development in patients aged between 0 and 8 years. The instrument is designed to specifically assess five main developmental areas: Locomotor, Personal-Social, Language, Eye-Hand Coordination, and Performance. For children between 2 and 8 years of age it also provides an additional scale of Practical Reasoning. For each subscale, mental age-equivalents and standardized developmental quotients (mean = 100, sd = 15) can be derived. Furthermore, a total mental age-equivalent and a General Developmental Quotient (mean = 100, sd = 15) are calculated. The instrument presents a satisfactory reliability in terms of total internal consistency (0.95). For what concerns the test-retest reliability, it appears to be satisfactory from the second year of life of the child, while results obtained administering the instrument during the first year do not show a good reliability of the instrument (32).

### Procedure

All procedures of this study were in accordance with the ethical code of the Italian Association of Psychology (AIP), with the last version of Declaration of Helsinki (33), and were approved by the Ethical Committee of the University of Trento (Protocol Number: 2020-042).

Data of each child were collected during the first diagnostic evaluation carried out by licensed psychologists at the ODFLab. To each child, the psychologist administered the Griffith Mental Development Scale-Edition Revised. Then, based on the child's developmental level and chronological age, the adequate ADOS-2 module was chosen to investigate ASD symptom severity. The clinical evaluation was recorded with bird's eye cameras. Subsequently, trained coders applied the coding system to the video recordings of the administration of the ADOS-2 using the Behavioral Observation Research Interactive Software (BORIS) (34), an open-source, time-constrained event logging software for video-coding developed by the University of Turin. Coders were blind to children's developmental measures and symptom severity. The coding window was set to 20 min for each videotape (30), selecting the activities that required or were more likely to involve dyadic interaction, e.g., Birthday Party, Bubble Play, Functional, and Symbolic Imitation.

If one or both the subjects were off camera during coding nothing was annotated until they were back on camera. If the coding window expired during an ongoing sharing of the action (SA) it was extended until the current interaction reached its end.

The socioeconomic status of the families was assessed through the four-factor index of social status (35).

### Statistical analysis

Data was aggregated at the session-level by a Python script, which allowed the extraction of the Units of Interaction (UIs) and the Shared Activity (SA). It also computed frequencies of the UIs, together with their proportions, durations, latencies. Moreover, success rates were extracted, that represent suitable parameters to study behavioral synchrony. The success rate refers to the ratio of the frequency of the starting codes when followed by a synchronous and adequate response, and the total frequency of the starting code. Success rates can also be computed in terms of efficacy of UIs in actually leading to the initiation of the interplay.

Statistical analysis was performed with R (36). Data were checked for normality through Shapiro–Wilk tests. We performed Welch's *t*-tests to study gender differences in chronological age, as it reduces the possibilities of Type 1 error and is particularly indicated when sample sizes are different, compared to Student's *t*-tests (37). Effect sizes were calculated using Cohen's D (38). We used linear models to study gender differences for each index, both behavioral descriptors and clinical measures. Age was always included in regressions to verify whether the differences emerged were dependent on it. We used the following formula:


lm(behavioral descriptor ~ sex+ age)

Finally, correlations between clinical measures and behavioral descriptors were measured using Pearson correlation coefficients or Kendall's Tau in case of violations of normality, since it is indicated as more robust than Spearman's Rho (39).

### Analytic plan

In the following section, we provided descriptive statistics of demographic data and clinical measures, the GMDS-ER and ADOS-2. Further, we studied the correlations between age and behavioral descriptors to better clarify the role of gender, since the female sample was significantly older than the male one. Linear regressions to study gender differences are presented in Section Gender differences in interaction behavior. Lastly, in Section Correlation analysis we presented correlations between behavioral descriptors and clinical measures, separately for females and males.

## Results

### Gender differences in age and clinical measures

Males and females presented significant age differences [*t*_(44)_ = −2.791, *p* = 0.008, d = 0.786], neither sex nor age were significant predictors of the general developmental quotient, as measured by the GMDS-ER, as the linear model was not significant.

The linear models performed to predict the scores of GMDS-ER and ADOS-2 scores, based on age and sex were not significant, except for the Performance subquotient of the GMDS-ER, which was significant [*F*_(2, 48)_ = 5.922, *p* = 0.005, *R*^2^ = 0.165], age was a significant predictor (β = −0.694, *p* = 0.023), but not sex (β = 10.767, *p* = 0.159). The model performed to predict the socioeconomic status based on sex and age was not significant. Further information is reported in Table 2.

**Table 2:** Demographic and clinical measures statistics.

	**Males** **Mean (sd) [Range]**	**Females** **Mean (sd) [Range]**	**Statistics**
Chronological age (months)**	38.962 (10.375) [22–57]	48.560 (13.863) [24–81]	*t*_(44)_ = −2.791, *p* = 0.008, *d* = 0.786
GMDS-ER general development quotient	72.115 (14.049) [48–98]	66.320 (23.637) [20–112]	*F*_(2, 48)_ = 1.757*, p* = 0.183, *R^2^* = 0.029
GMDS-ER locomotor development quotient	79.346 (20.433) [48–122]	72.040 (23.954) [20–127]	*F*_(2, 48)_ = 3.094, *p* = 0.054, *R^2^* = 0.078
GMDS-ER personal—social developmental quotient	69.615 (15.466) [40–93]	63.880 (24.624) [20–120]	*F*_(2, 48)_ = 0.956, *p* = 0.392, *R^2^* = −0.002
GMDS-ER language developmental quotient	59.231 (24.432) [24–116]	64.120 (32.751) [19–124]	*F*_(2, 48)_ = 0.216, *p* = 0.807, *R^2^* = −0.032
GMDS-ER eye-hand coordination developmental quotient	71.346 (16.587) [35–110]	63.880 (23.880) [20–106]	*F*_(2, 48)_ = 2.099, *p* = 0.134, *R^2^* = 0.042
GMDS-ER performance developmental quotient**	88.423 (22.422) [33–133]	71.000 (29.479) [20–136]	*F*_(2, 48)_ = 5.922, *p* = *0.005,* *R^2^* = *0.165*
ADOS-2 social affect	11.500 (3.023) [6–16]	12.040 (3.857) [4–19]	*F*_(2, 48)_ = 0.8483, *p* = 0.434, *R^2^* = −0.006
ADOS-2 restricted repetitive behaviors	3.462 (1.881) [0–7]	4.320 (1.865) [0–8]	*F*_(2, 48)_ = 1.748, *p* = 0.185, *R^2^* = 0.029
ADOS-2 total	14.962 (3.934) [8–23]	16.280 (4.551) [6–24]	*F*_(2, 48)_ = 0.747, *p* = 0.479, *R^2^* = −0.010
Socioeconomic status	37.167 (15.644) [13.5–63.0]	34.077 (15.241) [13.5–66.0]	*F*_(2, 34)_ = 0.292, *p* = 0.749, *R^2^* = −0.041

**p <0.01.

GMDS-ER, Griffiths mental development scales—edition revised; ADOS-2, Autism diagnostic observation schedule—second edition.

No significant correlations between age and behavioral descriptors emerged when correlations were performed within the two groups based on sex (see Table 3).

**Table 3:** Correlations among behavioral descriptors and age in male and female groups.

**Behavioral descriptors**	**Age (males)**	**Age (females)**
Rate of synchronous code pairs over the total code pairs	−0.35	0.06
Latency between the first code of the pair and the second	−0.03	−0.01
Rate of psychologist's proposal accepted by the child	0.02	−0.06
Latency between therapist proposal and child acceptance	0.02	0.16
Rate of child dysregulation signal caught by psychologist	−0.50	0.20
Percentage of Units of Interactions that led to a sharing	−0.04	0.15
Mean duration of the Shared Action	0.02	0.14
Sum of the durations of the shared actions	−0.05	0.22
Shared action complexity	0.39*	0.13
Success rate of psychologist widenings during a shared action	0.15	−0.08
Rate of interplays adequately concluded	0.07	0.15
Mean engagement displayed by the child during the Shared Action	−0.02	0.36

*p < 0.05.

### Gender differences in interaction behavior

Linear models were conducted to test gender differences with respect to behavioral descriptors. To account for the potential role of age, we always included it as a covariate in regressions.

The total number of events coded was predicted by sex but not by age and the model was significant. While, the overall coding time was not predicted by sex nor age.

Considering behavioral descriptors, the model related to the rate of synchronous code pairs over the total code pairs was significant [*F*_(2, 48)_ = 10.690, *p* <0.001, *R*^2^ = 0.279], in particular the synchronous behaviors were predicted by sex (β = −0.168, *p* <0.001), but not by age. Further, latencies between the first and the second code of the Interaction Units were predicted by sex (β = 0.154, *p* <0.001), but not by age, the model was significant [*F*_(2, 48)_ = 7.572, *p* = 0.001, *R*^2^ = 0.208].

Also, males presented a lower rate of accepted therapist's proposals. The model performed resulted to be significant [*F*_(2, 48)_ = 14.840, *p* <0.001, *R*^2^ = 0.356], with sex as a significant predictor (β = −0.224, *p* <0.001), but not age (β = −0.001, *p* = 0.532). Concluding, females presented faster responses to therapist's proposals when compared to males. The model resulted to be significant [*F*_(2, 48)_ = 7.768, *p* = 0.001, *R*^2^ = 0.213], with sex being a significant predictor (β = 0.250, *p* <0.001), but not age.

Considering the Action Sharings, males presented a lower percentage of Units of Interactions that led to a sharing. The model was significant [*F*_(2, 48)_ = 9.122, *p* <0.001, *R*^2^ = 0.245], with sex being a significant predictor (β = −0.255, *p* <0.001). Mean duration of the interplays was predicted by sex (β = −0.111, *p* = 0.011), and not by age. The model was significant [*F*_(2, 48)_ = 6.917, *p* <0.001, *R*^2^ = 0.194]. The overall time (s) spent engaged was predicted by sex (β = −279.431, *p* = 0.002), and not by age, the model resulted significant [*F*_(2, 48)_ = 7.635, *p* = 0.001, *R*^2^ = 0.210].

Sharing complexity was predicted by sex (β = −0.633, *p* = 0.035), and not by age. The model was significant [*F*_(2, 48)_ = 5.159, *p* = 0.009, *R*^2^ = 0.143].

Moreover, the success rate of psychologist's widenings accepted by the child was predicted by sex and not by age. The model was significant [*F*_(2, 47)_ = 5.654, *p* = 0.006, *R*^2^ = 0.160]. Females' interplays were also adequately concluded significantly more often. The model was significant [*F*_(2, 48)_ = 6.609, *p* = 0.003), *R*^2^ = 0.183], with sex being a significant predictor (β = −0.271, *p* = 0.004), but not age (β = 0.003, *p* = 0.469). Concluding, engagement was predicted by sex (β = −0.036, *p* <0.001), and not by age; the model was significant [*F*_(2, 48)_ = 24.24, *p* <0.001, *R*^2^ = 0.482] (see Table 4). Further information can be found in Supplementary Table 1.

**Table 4:** Gender differences in behavioral descriptors.

	**Gender differences**		
	**M** **Mean (sd) [Range]**	**F** **Mean (sd) [Range]**	**Statistics**
**Behavioral descriptors**
Total number of behaviors events coded**	64.846 (20.720) [34–134]	48.040 (15.296) [19–77]	*F*_(2, 48)_ = 7.295, *p* = 0.002, *R^2^* = 0.201
Coding time	1,261.541 (108.357) [1,078.376–1,510.134]	1,292.826 (178.540) [1,106.548–1,856.994]	*F*_(2, 48)_ = 0.325, *p* = 0.724, *R^2^* = −0.028
Rate of synchronous code pairs over the total code pairs***	0.675 (0.130) [0.405–1.000]	0.831 (0.114) [0.636–1.000]	*F*_(2, 48)_ = 10.690, *p* <0.001, *R^2^* = 0.279
Latency between the first code of the pair and the second**	2.366 (0.690) [1.453–3.872]	1.713 (0.702) [0.918–3.651]	*F*_(2, 48)_ = 7.572, *p* = 0.001, *R*^2^ = 0.208
Rate of psychologist's proposal accepted by the child***	0.697 (0.159) [0.333–1.000]	0.864 (0.153) [0.533–1.000]	*F*_(2, 48)_ = 14.840, *p* <0.001, *R^2^* = 0.356
Latency between therapist proposal and child acceptance**	2.387 (1.230) [1.010–6.173]	1.418 (0.629) [0.528–3.170]	*F*_(2, 48)_ = 7.768, *p* = 0.001, *R^2^* = 0.213
Percentage of units of Interactions that led to a sharing***	0.500 (0.182) [0.222–0.875]	0.745 (0.221) [0.333–1.000]	*F*_(2, 48)_ = 9.122, *p* <0.001, *R^2^* = 0.245
Mean duration of the shared action***	121.401 (89.105) [21–427]	204.878 (141.896) [82.143–669.000]	*F*_(2, 48)_ = 6.917, *p* <0.001, *R^2^* = 0.194
Sum of the durations of the shared actions**	578.846 (273.553) [84–1,249]	879.960 (282.571) [276–1,532]	*F*_(2, 48)_ = 7.635, *p* = 0.001, *R^2^* = 0.210
Shared action complexity**	2.254 (1.148) [0.000–4.800]	3.612 (2.152) [0.778–10.000]	*F*_(2, 48)_ = 5.159, *p* = 0.009, *R^2^* = 0.143
Success rate of psychologist widenings during a shared action**	0.532 (0.216) [0.167–1.000]	0.731(0.198) [0.250–1.000]	*F*_(2, 47)_ = 5.654, *p* = 0.006, *R^2^* = 0.160
Rate of interplays adequately concluded**	0.379 (0.294) [0–1]	0.653 (0.255) [0.167–1.000]	*F*_(2, 48)_ = 6.609, *p* = 0.003, *R^2^* = 0.183
Mean engagement displayed by the child during the shared action***	1.191 (0.172) [1.000–1.600]	1.843 (0.458) [1.000–2.500]	*F*_(2, 48)_ = 24.24, *p* <0.001, *R^2^* = 0.482

**p <0.01, ***p < 0.001.

### Correlation analysis

Considering clinical measures and behavioral descriptors, the success rate of psychologist proposals accepted by the child positively correlated with the GMDS-ER Language Quotient in males [*r*_(24)_ = 0.41, *p* = 0.038], and in females (τ = 0.40, *p* = 0.047). Further, it negatively correlated with the ADOS-2 Social Affect score (τ = −0.36, *p* = 0.020) in females.

Considering the interplays, the number of Action Sharings positively correlated with the Performance Quotient in males [*r*_(24)_ = 0.41, *p* = 0.039], but not in females [*r*_(23)_ = −0.28, *p* = 0.174]. The mean duration of the shared actions during the interaction positively correlated with Personal-Social (τ = 0.41, *p* = 0.004) and Language (τ = 0.41, *p* = 0.004) Quotients, and it negatively correlated with Social Affect (τ = −0.32, *p* = 0.03) in females, but not in males. A lower severity in the Social Affect domain was related to a greater engagement displayed during the interplays in females [*r*_(23)_ = −0.50, *p* = 0.011], but not in males. Further, Personal-Social (τ = 0.37, *p* = 0.011) and Language (τ = 0.36, *p* = 0.014) Quotients were positively correlated to the complexity of the Shared Actions in females, but not in males.

Finally, the rate of interplays adequately ended by the child positively correlated with Personal-Social (τ = 0.46, *p* = 0.003) and Language (τ = 0.49, *p* = 0.002) Quotients in females, but not in males. For further information, see Table 5.

**Table 5:** Correlations among behavioral descriptors and clinical measures for the females and males group.

	**Males**				**Females**			
	**QB**	**QC**	**QE**	**SA**	**QB**	**QC**	**QE**	**SA**
Success rate of psychologist proposals	0.17	0.41*	0.11	**–**0.22	0.29	0.40*	0.13	0.51**
Number of Shared Actions	−0.002	−0.17	0.41*	−0.006	−0.35	−0.39	−0.28	0.13
Mean duration of the shared actions	0.12	0.24	−0.19	0.05	0.41**	0.41**	0.22	−0.32*
Shared action complexity	0.07	−0.05	−0.09	−0.09	0.37*	0.36*	0.28	−0.26
Mean engagement displayed by the child during the shared action	0.10	−0.17	−0.06	0.02	0.35	0.37	0.15	−0.50*
Rate of interplays adequately ended by the child	0.14	0.07	0.17	−0.33	0.46**	0.49**	0.13	−0.26

*p <0.05; **p <0.01.

QB, Personal-Social development Quotient; QC, Language development quotient; QE, Performance development quotient. These quotients are calculated with using the Griffiths Mental Development Scales—Edition Revised (GMDS-ER). SA, Social affect score measured by the Autism Diagnostic Observation Schedule—Second Edition (ADOS-2), the higher it is the higher is the presence of symptoms in this area.

## Discussion

The purpose of the present study was deepen the knowledge on gender differences in the interaction profiles of pre-schoolers with ASD. To achieve our aims and to overcome previous studies limitation, which was represented by the focus on broader constructs (18), we investigated Interpersonal Synchrony using an observational coding system that allowed to objectively study the bidirectional interactive behaviors of a dyad.

### Gender differences

With respect to the general purpose of our study, we found gender differences in the age of first diagnosis, as females were significantly older than males, in line with current literature which highlights that girls with ASD are under- and later- recognized (4, 35, 40). This can be partly justified by the analysis of the behavioral descriptors, which indeed highlighted better social interaction skills in girls. More specifically, we confirmed our hypothesis as we found that females tend to respond more often and quicker to the psychologist. Further, they presented longer, more complex and engaging interplays, and better use of their communicative abilities to adequately conclude the interaction, compared to males with ASD. In line with this, a recent review highlighted that females with ASD present significantly better social interaction and socio-communication abilities than males with ASD, and these differences tend to be more visible when considering narrow constructs (14). Considering that IS deeply impacts social perception (41), females' interaction profile, characterized by better Interpersonal Synchrony, with higher involvement and responsiveness during the interplay, might alter how the child is perceived by others, ultimately resulting in less concern raised for females' behaviors. Hence, caregivers and teachers might not be trained to identify nuanced presentations of ASD socio-communicative symptoms and therefore they might be misled by apparent greater social competencies and social adaptability (7). It has been also recently suggested that educators may be biased and less sensitive to ASD presentation in girls (42). This, combined with the influence of the male bias and gender norms might result in a later referral of the child to professionals, resulting in far-reaching consequences. Hence, despite presenting better interaction features compared to males with ASD, females still present difficulties related to ASD core symptoms, as emerged by the absence of significant sex effect in the ADOS-2 scores. This is in line with a recent meta-analysis that showed the absence of gender differences in the social and communication domain evaluated with clinical measures (ADOS and the Autism Diagnostic Interview—Revised) (43). Considering that current literature focuses on broader domains, a possible avenue for future research could be to study the different subdomains evaluated by clinical tools to highlight potential gender differences.

Further, future research should deepen the knowledge on the role of IS in the late diagnosis of ASD in females; if the child accesses to intervention earlier, developmental outcomes will improve (44), as age at the beginning of treatment is a strong predictor of social communication outcomes (45).

### Relationship between behavioral descriptors and clinical measures

Interestingly, the behavioral descriptors that presented gender differences in our sample also differently correlated with clinical measures in males and females, indicating the relevance of the indexes studied.

Our findings suggested that when engaging in interplays, females and males differently rely on their abilities. Males' successful interaction depends more on cognitive skills, specifically performance abilities, while females appear to rely more on social skills. Both males and females rely on language skills in order to respond to others' proposals.

Our results also showed that Interpersonal Synchrony, longer, more complex and engaging interplays, are associated with language acquisition and personal-social development in females. As we didn't find significant correlations in males in this sense, it is possible to hypothesize that child development, in presence of ASD symptoms that hinder socio-communicative abilities, relies on potentially different processes that, in turn, may depend on child gender. Understanding the mechanisms that scaffold the emergence of skills in different developmental domains is crucial, as they might have an impact on intervention response and personalization. In fact, our results suggested that practitioners should consider gender differences and the specific interaction profiles not only during the diagnostic assessments, but also when defining intervention goals and strategies. It can also be suggested that studying gender specific differences may be crucial to treatment optimization and requires further research.

### Limitations and future directions

Our study presents some constraints. The main one is represented by the small sample size, which hinders the generalizability of our results; hence, future studies should be conducted on larger samples. Another limitation is represented by the lack of a comparison group constituted by neurotypical individuals which could be relevant in understanding whether the differences emerged can be ascribable to ASD or to child development at a more general level. Despite that, being aware of the females with ASD's interaction profile is crucial when warning signs need to be identified by caregivers and teachers, as well as during the diagnostic process, since many clinicians can be partly influenced by the male bias and other contextual biases (8, 46, 47).

Further, our research considers just the assessment at the time of the first diagnosis. More research should focus on analyzing different time points in order to understand if and how IS changes during intervention and to learn more on how it can impact on treatment response and child development.

Our research also highlighted a greater engagement of females during the interplay. As the Interpersonal Synchrony coding scheme presents just one index that evaluates the level of participation and shared affect displayed by the child, future research should combine it with measures that study involvement and responsiveness from a qualitative point of view. This would help researchers to better understand gender differences and factors that might play a role in the perception that adults have of the child.

A final limitation is represented by the videos we analyzed, as we studied the ADOS-2 administration, which is a more structured context with several activities that have to be completed, we could not study in depth gender differences in child intentionality signals and proposals. In light of the difficulties presented by children with ASD in intentionality and considering the relevance of this index, future research should examine free dyadic play interactions.

## Conclusions

This study highlighted that focusing on narrower constructs may be crucial to detect gender differences in pre-schoolers with ASD and Interpersonal Synchrony could be a relevant construct.

Accordingly with clinical experience, we found that females present better IS abilities as they appeared more responsive and involved in the social exchange. Greater abilities might influence others' perception of social connectedness and child's skills, especially with nuanced ASD symptom manifestations. This might have an impact on the child referral and access to services, and may explain, at least partly, the late-diagnosis of females that emerged from both our study and current literature.

Further, the indexes that showed gender difference differentially correlated with clinical measures of child development and ASD symptomatology, suggesting differences in the abilities males and females rely on in order to successfully interact with others. Better understanding females' interaction profiles may be crucial for tailoring the assessment process and in defining adequate intervention strategies. Nevertheless, our results suggested that IS differently scaffold child development in males and females. Understanding these specific mechanisms may have relevant implications for intervention.

## Data availability statement

The datasets presented in this article are not readily available because of privacy reasons. The data that support the findings of this work are available upon reasonable request from the corresponding author. The observational coding system is available under request to the corresponding author. Requests to access the datasets should be directed to EP, eleonora.paolizzi@unitn.it.

## Ethics statement

The studies involving human participants were reviewed and approved by Research Ethics Committee of the University of Trento. Written informed consent to participate in this study was provided by the participants' legal guardian/next of kin.

## Author contributions

EP, GB, AB, and PV contributed to the study conception and design, designed the statistical methodology, and writing—review and editing. Data collection was performed by AB. Material preparation was performed by EP and GB. EP annotated the videos, as expert and trained observer. Statistical analysis was performed by EP. The first draft of the manuscript was written by EP and GB. All authors commented on previous versions of the manuscript, contributed to review and edit the manuscript, and read and approved the final manuscript.

## Conflict of interest

The authors declare that the research was conducted in the absence of any commercial or financial relationships that could be construed as a potential conflict of interest.

## Publisher's note

All claims expressed in this article are solely those of the authors and do not necessarily represent those of their affiliated organizations, or those of the publisher, the editors and the reviewers. Any product that may be evaluated in this article, or claim that may be made by its manufacturer, is not guaranteed or endorsed by the publisher.
