# Supplementary material for: Interpersonal synchrony: Interaction variables and gender differences in preschoolers with ASD
Source: Front Psychiatry. 2022 Sep 14;13:1009935. doi: 10.3389/fpsyt.2022.1009935 (PMC9515646; doi:10.3389/fpsyt.2022.1009935)
Supplement: Supplementary file 1 [file Table_1.DOCX]

***Supplementary Material***

**Table 1.** Linear models performed to study gender differences in behavioral descriptors considering the potential influence of age.

| **Gender Differences** | | | |
| --- | --- | --- | --- |
|  | Sex (β) | Age (β) | **Statistics** |
| Behavioral Descriptors | | | |
| Total number of behaviors events coded** | β=0.192, p=0.014 | β=-0.005, p= 0.098 | F(2,48)= 7.295, p=0.002, R²=0.201 |
| Coding time | β<0.001, p=0.714 | β<0.001, p=0.599 | *F(2,48)= 0.325, p=0.724,* R²=-0.028 |
| Rate of synchronous code pairs over the total code pairs *** | β=-0.168, p<0.001 | β=-0.001; p=0.383 | *F(2,48)=10.690, p<0.001,* R²=*0.279* |
| Latency between the first code of the pair and the second** | β=0.154, p<0.001 | β=0.002; p=0.470 | *F(2,48)= 7.572, p=0.001,* R²=*0.208* |
| Rate of psychologist’s proposal accepted by the child*** | β=-0.224, p<0.001 | β=-0.001, p=0.532 | *F(2,48)= 14.840, p<0.001,* R²=*0.356* |
| Latency between therapist proposal and child acceptance** | β=0.250, p<0.001 | β=0.001, p=0.720 | *F(2,48)=7.768, p=0.001,* R²=0.213 |
| Percentage of Units of Interactions that led to a sharing*** | β=-0.255, p<0.001 | β=0.001, p=0.533 | *F(2,48)=9.122, p<0.001,* R²=0.245 |
| Mean duration of the Shared Action*** | β=-0.111, p=0.011 | β=0.002, p=0.158 | *F(2,48)=6.917, p<0.001,* R²=*0.194* |
| Sum of the durations of the Shared Actions** | β=-279.431, p=0.002 | β=2.259, p=0.493 | *F(2,48)=7.635, p=0.001,* R²=*0.210* |
| Shared Action complexity** | β=-0.633, p=0.035 | β=0.016, p=0.171 | *F(2,48)=5.159, p=0.009,* R²=*0.143* |
| Success rate of psychologist widenings during a Shared Action** | β=-0.194, p=0.004 | (β=0.0004, p=0.835) | *F(2,47)=5.654, p=0.006,* R²=*0.160* |
| Rate of interplays adequately concluded** | β=-0.271, p=0.004 | β=0.003, p=0.469 | *F(2,48)=6.609, p=0.003, R²=0.183* |
| Mean engagement displayed by the child during the Shared Action*** | β=-0.036, p<0.001 | β=0.0003, p=0.224 | *F(2,48)=24.24, p<0.001, R²=0.482* |

** p < 0.05; ** p < 0.01, ***p < 0.001*

β, estimate.
